# One-Pot Synthesis of Novel 2,3-Dihydro-1*H*-indazoles

**DOI:** 10.3390/molecules16119553

**Published:** 2011-11-16

**Authors:** Gary W. Breton, Antonio J. Lepore

**Affiliations:** Department of Chemistry, Berry College, PO Box 495016, Mount Berry, GA 30101, USA

**Keywords:** 2,3-dihydro-1*H*-indazole, synthesis, heterocycle, indazoles, copper coupling

## Abstract

A copper(I)-mediated one-pot synthesis of 2,3-dihydro-1*H*-indazole heterocycles has been developed. This synthetic route provides the desired indazoles in moderate to good yields (55%–72%) which are substantially better than those achievable with an alternative two-step reaction sequence. The reaction is tolerant of functionality on the aromatic ring.

## 1. Introduction

**Figure 1:**
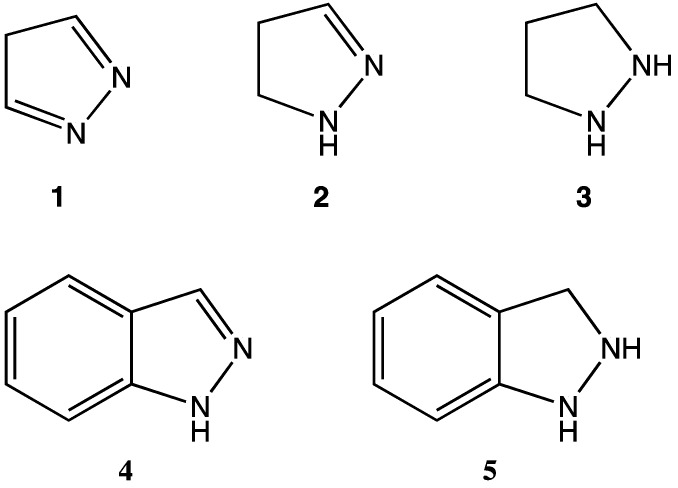
Structures of select 1,2-dinitrogen heterocycles.

Heterocycles are important structural units found in a wide range of biologically active compounds [1]. There have been many calls for the synthesis of novel heterocyclic systems to be used as building blocks for the next generation of pharmaceuticals [1,2,3,4]. One subclass of particularly active heterocycles are those bearing 1,2-dinitrogen substitution. For example, substituted derivatives of the pyrazole (**1** in Figure 1), pyrazoline (**2**), and pyrazolidine (**3**) ring systems are of pharmaceutical interest for their demonstrated antibacterial, antidepressant, and/or anti-inflammatory activities (among others) [4,5,6]. Additionally, the fused aromatic 1*H*-indazole (**4**) is well recognized for its antihypertensive and anticancer properties [7]. Surprisingly, however, the 2,3-dihydro-1*H*-indazole moiety (**5** in Figure 1) remains virtually unknown and untested, despite its obvious structural similarities to both the pyrazolidine (**3**) and 1*H*-indazole (**4**) ring systems.

**Scheme 1:**
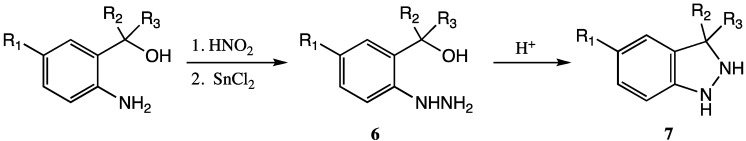
Synthesis of substituted derivatives of 2,3-dihydro-1*H*-indazoles **7** via acid-catalyzed ring-closure.

We are aware of only a handful of studies that have addressed the synthesis of derivatives of indazoles **5** [8,9,10,11,12,13]. Zenchoff reported on their synthesis via acid-catalyzed S_N_1-type ring closure of substituted 2-hydroxymethyl phenylhydrazines **6** to form indazoles **7** (Scheme 1) [8]. Overall yields of approximately 30% were reported for the combined two-step method.

Additionally, there have been a few studies in which formation of derivatives of indazole **5** have been observed as a result of trapping experiments on reactive intermediates [9,10,11]. However, other than Zenchoff’s limited work, we are unaware of the development of any general routes for the synthesis of this class of heterocycles.

In addition to being interesting compounds in their own right, 2,3-dihydro-1*H*-indazoles **5** have also served as synthetic intermediates for their more thoroughly-studied counterparts, the 1*H*-indazoles **4** [8]. Thus, development of synthetic routes towards the synthesis of indazoles **5** similarly creates novel routes for the synthesis of indazoles **4**.

The sparse number of investigations into the synthesis of this class of heterocycles is, therefore, quite surprising given that 2,3-dihydro-1*H*-indazoles have such strong potential for biological activity and as convenient synthetic intermediates. Detailed investigations of their properties, however, demand robust synthetic methods for their preparation. Herein, we describe our initial investigations towards the synthesis of this understudied class of heterocyclic compounds.

## 2. Results and Discussion

**Scheme 2:**
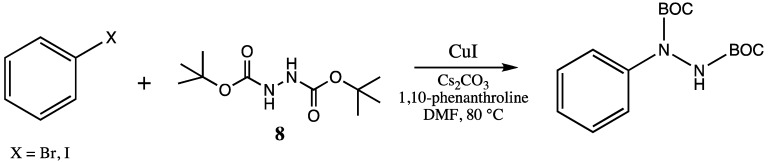
Ullman-type coupling of hydrazine **8** to halogenated aromatics.

**Scheme 3:**
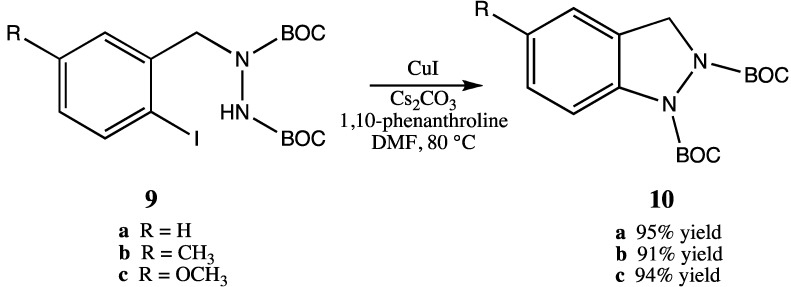
Intramolecular coupling of hydrazines **9** to form bis-BOC protected indazoles **10**.

Ullman-type copper(I)-mediated coupling of bis-BOC protected hydrazine **8** to aryl and vinyl halides in DMF using CuI, 1,10-phenanthroline and Cs_2_CO_3_ is a well-established synthetic procedure (see Scheme 2) [14,15]. We initially envisioned the synthesis of bis-BOC protected 2,3-dihydro-1*H*-indazoles **10** via intramolecular coupling of the free N-H bond of hydrazines **9** at the iodo-substituted carbon atom to form the five-membered indazole nucleus (Scheme 3). Indeed, this proved to be an excellent method for the synthesis of substituted indazoles, providing high yields of **10** (Scheme 3).

**Scheme 4:**
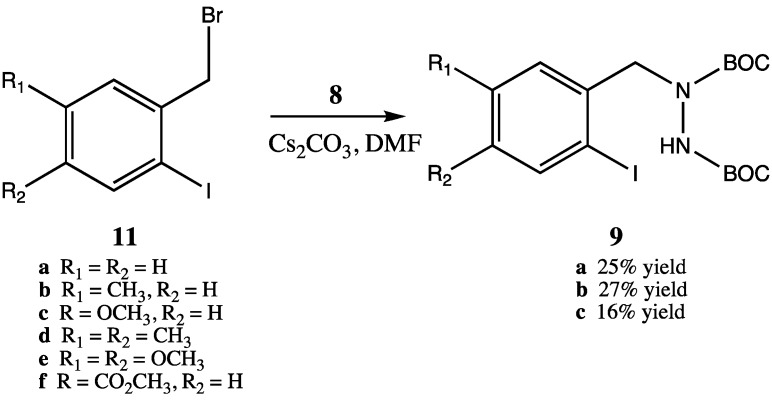
Synthesis of monoalkylated bis-BOC protected hydrazines **9**.

Unfortunately, however, synthesis of the required trisubstituted hydrazine starting materials **9** proved to be problematic (Scheme 4). Although we utilized reaction conditions optimized by Rasmussen for selective monoalkylation of **8** [16], only poor yields of monoalkylated product were obtained (*i.e.*, compounds **10a–c** were obtained in 25%, 27% and 16% yields, respectively). Surprisingly, bisalkylation of hydrazine **8** was the preferred reaction route. We attempted to increase the yield of monoalkylated product by increasing the amount of starting **8** relative to starting benzyl bromides **11**, but this led to difficulties in separation of the product from excess **8**. Additionally, other than **11a**, which was commercially available, the required substituted *ortho*-iodobenzyl bromides (compounds **11b–f**) needed to be synthesized from the corresponding benzylic alcohols via a two step process of iodination of the aromatic ring (silver CF_3_CO_2_Ag/I_2_) followed by bromination at the benzylic alcohol position (PBr_3_). Thus, although the intramolecular coupling reactions afforded high yields (Scheme 3), the effective yields from the synthetically expensive ortho-iodobenzyl bromides were unsatisfactorily low.

To bypass the problem of dialkylation, we considered the possibility of developing a one-pot procedure in which copper-mediated coupling of the initially formed monoalkylated hydrazines **9** to form indazoles **10** might be able to compete with the complicating dialkylation process. A mixture of **8** and *ortho*-iodobenzyl bromide **11a** was added slowly, via syringe pump (over a period of 7 h), to a pre-heated stirring mixture of CuI, 1,10-phenanthroline and Cs_2_CO_3_. After 24 h, we were gratified to note that the only major product detected by TLC analysis of the crude reaction mixture was the desired indazole **10a**. Reaction workup, followed by column chromatography afforded **10a** in 60% yield, a substantial increase from the effective 22% yield obtained via the two-step process (*i.e.*, **11a**→**9a**→**10a**) starting from the *ortho*-iodobenzyl bromide **11a**. In addition, this procedure avoided the need for purification of the intermediate hydrazine **9a**. Similarly, indazoles **9b** and **9c** were formed in 60% yields utilizing the one-pot procedure rather than the effective 25% and 15% yields, respectively, from the corresponding two-step procedures.

Given the success of this one-pot procedure, we subjected a number of substituted ortho-iodobenzyl bromides to the same reaction conditions. The yields were consistent at ~60% (Table 1), and the products were obtained in pure form following column chromatography. The reaction was tolerant of electron-donating (*i.e.*, OCH_3_) and electron-withdrawing (*i.e.*, CO_2_CH_3_) groups.

**Table 1:** One-pot synthesis of substituted bis-BOC protected indazoles **10** starting from *ortho*-iodo benzylbromides **11**. 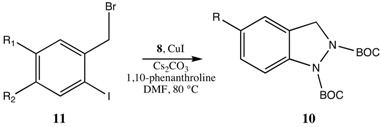

Entry	Substrate	Indazole Product	Yield (%)
1	**11a**	**10a**	60
2	**11b**	**10b**	60
3	**11c**	**10c**	60
4	**11d**	**10d**	55
5	**11e**	**10e**	72
6	**11f**	**10f**	62

## 3. Experimental

### 3.1. General

All chemicals and solvents were used as received (Aldrich, St Louis, MO, USA) Anhydrous DMF was kept under nitrogen and sealed with a septum. Column chromatography was performed using 230–400 mesh silica gel 60. NMR spectra were recorded on a Varian 60 MHz instrument in CDCl_3_ as solvent, unless otherwise indicated, and referenced relative to TMS (0.0 PPM). Combustion analysis was performed by Micro Analysis Inc. (Wilmington, DE, USA). Other than unsubstituted 2-iodobenzyl bromide **11a**, which was commercially available (Aldrich), benzyl bromides **11b–f** were synthesized via standard aromatic iodination (I_2_, CF_3_CO_2_Ag) followed by bromination of the benzylic alcohol (PBr_3_) following procedures described in the literature [17].

### 3.2. Synthesis of Alkylated Monoalkylated Hydrazines ***9a–c***

*Representative Procedure*: *Preparation of 1,2-di-tert-Butyl 1-(2-iodobenzyl)-1,2-hydrazinedicarboxylate* (**9a**) To a solution of di-*tert*-butyl hydrazodiformate (**8**, 0.71 g, 3.06 mmol) in anhydrous DMF (15 mL) was added Cs_2_CO_3_ (2 g, 2 equiv.) followed immediately by 2-iodobenzyl bromide (**11a**, 1 g, 3.37 mmol, 1.1 equiv.). The mixture was stirred for 2 h, after which time TLC indicated consumption of the bromide. The mixture was diluted with H_2_O (25 mL) and washed with EtOAc (3 × 25 mL). The combined EtOAc layers were backwashed with brine (3 × 25 mL), dried and concentrated. Column chromatography (SiO_2_, 4:1 hexanes/EtOAc as eluent) afforded 0.32 g (23% yield) of **9a** as a white solid. ^1^H-NMR δ 7.82 (d, *J* = 7.4 Hz, 1H), 7.34–6.79 (m, 3H), 6.45 (br s, NH, 1H), 4.71 (s, 2H), 1.48 (s, 9H), 1.45 (s, 9H). ^13^C-NMR δ 155.3, 155.0, 139.5, 139.3, 129.7, 129.1, 128.3, 99.0, 81.5, 81.1, 58.1, 28.2. Anal. Calcd. for C_17_H_25_N_2_O_4_I: C, 45.53; H, 5.62; N, 6.25; H. Found: C, 45.61; H, 5.56; N, 6.12.

*1,2-Di-tert-butyl 1-(2-iodo-5-methylbenzyl)-1,2-hydrazinedicarboxylate* (**9b**). Compound **9b** (0.46 g, 27% yield) was isolated as a white solid following the representative procedure described above starting with 2-iodo-5-methylbenzyl bromide (**11b**, 1.1 g, 3.37 mmol). ^1^H-NMR δ 7.64 (d, *J* = 8.0 Hz, 1H), 7.10 (d, *J* = 1.7 Hz, 1H), 6.74 (dd, *J* = 8.0, 1.7 Hz, 1H), 6.67 (br s, NH, 1H), 4.67 (s, 2H), 2.27 (s, 3H), 1.48 (s, 9H), 1.44 (s, 9H). ^13^C-NMR δ 155.1, 154.7, 138.9, 138.6, 137.8, 130.2, 129.9, 94.7, 81.1, 80.6, 57.4, 27.9, 20.7. Anal. Calcd. for C_18_H_27_N_2_O_4_I: C, 46.74; H, 5.89; N, 6.06; H. Found: C, 46.61; H, 5.84; N, 5.96.

*1,2-Di-tert-butyl 1-(2-iodo-5-methoxybenzyl)-1,2-hydrazinedicarboxylate* (**9c**). Compound **9c** (0.24 g, 16% yield) was isolated as a thick colorless liquid following the representative procedure described above starting with 2-iodo-5-methoxybenzyl bromide (11c, 1.1 g, 3.42 mmol). ^1^H-NMR δ 7.67 (d, *J* = 8.7 Hz, 1H), 6.93 (d, *J* = 3.0 Hz, 1H), 6.55 (dd, *J* = 8.7, 3.0 Hz, 1H), 6.45 (br s, NH, 1H), 4.66 (s, 2H), 3.76 (s, 3H), 1.48 (s, 9H), 1.45 (s, 9H). ^13^C-NMR δ 160.1, 155.3, 154.9, 140.3, 140.0, 115.6, 115.1, 87.1, 81.6, 81.2, 58.0, 55.3, 28.2. Anal. Calcd. for C_18_H_27_N_2_O_5_I: C, 45.18; H, 5.86; N, 5.69; H. Found: C, 43.81; H, 5.51; N, 5.55.

### 3.3. Synthesis of 1,2-Di-tert-butyl 1H-indazole-1,2-(3H)-dicarboxylates ***10a–c*** via Intramolecular Cyclization

*Representative Procedure*: *Preparation of* *1,2-di-tert-Butyl 5-methyl-1H-indazole-1,2-(3H)-di-carboxylate* (**10b**). A mixture of **9b** (0.31 g, 6.7 mmol), CuI (0.13 g, 1 equiv.), 1,10-phenanthroline (0.12 g, 1 equiv.) and Cs_2_CO_3_ (0.33 g, 1.5 equiv.) in anhydrous DMF (5 mL) was stirred under an atmosphere of N_2_ at 80 °C for 24 h. The solution was cooled, and filtered through a short column of Celite which was rinsed thoroughly with EtOAc (100 mL). The resulting mixture was filtered to remove an insoluble precipitate, concentrated to a thick brown oil, and subjected to column chromatography (SiO_2_ using 4:1 hexanes/EtOAc as eluent) to afford **10b** (0.20 g, 91% yield) as a thick colorless oil. ^1^H-NMR δ 7.42 (d, *J* = 8.8 Hz, 1H), 7.10–6.99 (m, 2H), 5.18–4.45 (m, 2H), 2.32 (s, 3H), 1.56 (s, 9H), 1.50 (s, 9H). ^13^C-NMR δ 156.4, 153.0, 138.0, 134.0, 128.3, 122.6, 115.9, 82.2, 81.9, 51.5, 28.2, 20.9. The glassy nature of this product prevented us from obtaining a satisfactory C,H,N analysis. Evidence of purity is provided by the NMR spectra given in the Supplementary Material.

*1,2-Di-tert-butyl 1H-indazole-1,2-(3H)-dicarboxylate* (**10a**). Compound **10a** (0.21 g, 95% yield) was isolated as a white solid following the representative procedure starting with **9a** (0.31 g, 0.68 mmol). ^1^H-NMR δ 7.64–7.07 (m, 4H), 5.22–4.51 (m, 2H), 1.57 (s, 9H), 1.51 (s, 9H). ^13^C-NMR δ 156.4, 152.8, 140.2, 128.2, 127.8, 124.2, 122.0, 116.1, 82.4, 82.0, 51.6, 28.2. Anal. Calcd. for C_17_H_24_N_2_O_4_: C, 63.72; H, 7.55; N, 8.75; H. Found: C, 63.81; H, 7.56; N, 8.56.

*1,2-Di-tert-butyl 5-methoxy-1H-indazole-1,2-(3H)-dicarboxylate* (**10c**). Compound **10c** (0.11 g, 94% yield) was isolated as a white solid following the representative procedure starting with **9c** (0.16 g, 0.34 mmol). ^1^H-NMR δ 7.51–7.35 (m, 1H), 6.88–6.73 (m, 2H), 5.15–4.46 (m, 2H), 3.78 (s, 3H), 1.56 (s, 9H), 1.51 (s, 9H). ^13^C-NMR δ 157.1, 156.3, 153.2, 133.8, 129.6, 116.9, 113.0, 108.0, 82.1, 81.8, 55.6, 51.6, 28.2. Anal. Calcd. for C_18_H_26_N_2_O_5_: C, 61.68; H, 7.48; N, 8.00; H. Found: C, 61.70; H, 7.47; N, 7.87.

### 3.4. Synthesis of 1,2-Di-tert-butyl 1H-indazole-1,2-(3H)-dicarboxylates ***10a–f*** via the One-Pot Procedure

*Representative Procedure*: *Preparation of 1,2-di-tert-Butyl 5-methoxy-1H-indazole-1,2-(3H)-di-carboxylate* (**10c**). To a stirring mixture of CuI (0.58 g, 1 equiv.), 1,10-phenanthroline (0.55 g, 1 equiv.) and Cs_2_CO_3_ (2 g, 2 equiv.) in anhydrous DMF (10 mL) under N_2_ preheated to 80 °C a solution of 2-iodo-5-methoxybenzyl bromide **11c** (1 g, 3.06 mmol) and di-*tert*-butyl hydrazodiformate (**8**, 1.06 g, 1.5 equiv.) in anhydrous DMF (7 mL) was added via syringe pump at a rate of 1 mL/h. The entire reaction (including injection time) was allowed to proceed for 24 h, after which time it was cooled and filtered through a short column of Celite which was rinsed thoroughly with EtOAc (150 mL). The resulting EtOAc mixture was filtered to remove an insoluble precipitate, concentrated to a thick brown oil, and subjected to column chromatography (SiO_2_ using 4:1 hexanes/EtOAc as eluent) to afford 0.64 g (60% yield) of **10c** as a white crystalline solid.

*1,2-Di-tert-butyl 1H-indazole-1,2-(3H)-dicarboxylate* (**10a**). Compound **10a** (0.97 g, 60% yield) was isolated as a white solid following the representative procedure starting with 2-iodobenzyl bromide (**11a**, 1.50 g, 5.05 mmol).

*1,2-Di-tert-butyl 5-methyl-1H-indazole-1,2-(3H)-dicarboxylate* (**10b**). Compound **10b** (0.65 g, 60% yield) was isolated as a thick colorless liquid following the representative procedure starting with 2-iodo-5-methylbenzyl bromide (**11b**, 1.0 g, 3.22 mmol).

*1,2-Di-tert-butyl 5,6-dimethyl-1H-indazole-1,2-(3H)-dicarboxylate* (**10d**). Compound **10d** (0.59 g, 55% yield) was isolated as a thick colorless liquid following the representative procedure starting with 2-iodo-4,5-dimethylbenzyl bromide (11d, 1.0 g, 3.10 mmol). ^1^H-NMR δ 7.36 (s, 1H), 6.96 (s, 1H), 5.16–4.43 (m, 2H), 2.27 (s, 3H), 2.23 (s, 3H), 1.57 (s, 9H), 1.50 (s, 9H). ^13^C-NMR δ 156.5, 153.1, 138.4, 136.0, 132.4, 125.6, 122.9, 117.2, 82.0, 81.7, 51.5, 28.2, 20.1, 19.4. The glassy nature of this product prevented us from obtaining a satisfactory C,H,N analysis. Evidence of purity is provided by the NMR spectra given in the Supplementary Material.

*1,2-Di-tert-butyl 5,6-dimethoxy-1H-indazole-1,2-(3H)-dicarboxylate* (**10e**). Compound **10e** (0.85 g, 72% yield) was isolated as a thick colorless liquid following the representative procedure starting with 2-iodo-4,5-dimethoxybenzyl bromide (**11e**, 1.1 g, 3.01 mmol). ^1^H-NMR δ 7.36, 7.21 (s, 1H), 6.75 (s, 1H), 5.15-4.47 (m, 2H), 3.90 (s, 3H), 3.85 (s, 3H), 1.57 (s, 9H), 1.51 (s, 9H). ^13^C-NMR δ 156.5, 153.2, 149.0, 146.7, 134.0, 119.0, 105.9, 101.4, 82.1, 81.8, 56.5, 56.1, 51.8, 28.2. The glassy nature of this product prevented us from obtaining a satisfactory C,H,N analysis. Evidence of purity is provided by the NMR spectra given in the Supplementary Material.

*1,2-Di-tert-butyl 5-carbomethoxy-1H-indazole-1,2-(3H)-dicarboxylate* (**10f**). Compound **10f** (0.43 g, 62% yield) was isolated as a thick colorless liquid following the representative procedure starting with 2-iodo-5-carbomethoxybenzyl bromide (**11f**, 0.65 g, 1.83 mmol). ^1^H-NMR δ 8.08-7.89 (m, 2H), 7.55 (d, *J* = 8.3Hz, 1H), 5.02–4.75 (m, 2H), 3.90 (s, 3H), 1.58 (s, 9H), 1.51 (s, 9H). ^13^C-NMR δ 166.3, 156.3, 151.9, 143.8, 130.3, 128.4, 126.1, 123.7, 115.2, 83.0, 82.4, 52.0, 51.5, 28.1. Anal. Calcd. for C_19_H_26_N_2_O_6_: C, 60.29; H, 6.93; N, 7.41; H. Found: C, 60.35; H, 6.96; N, 7.15.

## 4. Conclusions

In summary, a convenient synthesis of substituted 2,3-dihydro-1*H*-indazoles has been developed. This one-pot procedure provides indazoles at considerably higher yields than the corresponding two-step process. This constitutes one of the first general synthetic methods for this class of heterocyclic compounds.

## Supplementary Materials

Supplementary Materials can be accessed on: http://www.mdpi.com/1420-3049/16/11/9553/s1.
