# Supplementary material for: One-Pot Synthesis of Novel 2,3-Dihydro-1H-indazoles
Source: Molecules. 2011 Nov 16;16(11):9553–61. doi: 10.3390/molecules16119553 (PMC6264256; doi:10.3390/molecules16119553)
Supplement: Supplementary file 1 [file molecules-16-09553-s001.docx]

One-Pot Synthesis of Novel 2,3-Dihydro-1*H*-indazoles

**Gary W. Breton * and Antonio J. Lepore**

Department of Chemistry, Berry College, Mount Berry, GA 30149, USA

**Supplementary Materials**

1. ^1^H-NMR spectrum of 1,2-di-*tert*-butyl 5-methyl-1*H*-indazole-1,2-(3*H*)-dicarboxylate (**10b**)

2. ^13^C-NMR spectrum of 1,2-di-*tert*-butyl 5-methyl-1*H*-indazole-1,2-(3*H*)-dicarboxylate (**10b**)

3. ^1^H-NMR spectrum of 1,2-di-*tert*-butyl 5,6-dimethyl-1*H*-indazole-1,2-(3*H*)-dicarboxylate (**10d**)

4. ^13^C-NMR spectrum of 1,2-di-*tert*-butyl 5,6-dimethyl-1*H*-indazole-1,2-(3*H*)-dicarboxylate (**10d**)

5. ^1^H-NMR spectrum of 1,2-di-*tert*-butyl 5,6-dimethoxy-1*H*-indazole-1,2-(3*H*)-dicarboxylate (**10e**)

6. ^13^C-NMR spectrum of 1,2-di-*tert*-butyl 5,6-dimethoxy-1*H*-indazole-1,2-(3*H*)-dicarboxylate (**10e**)

**7. ^1^H NMR spectrum of** **1,2-di-*tert*-butyl 5-methyl-1*H*-indazole-1,2-(3*H*)-dicarboxylate (10b)**

**
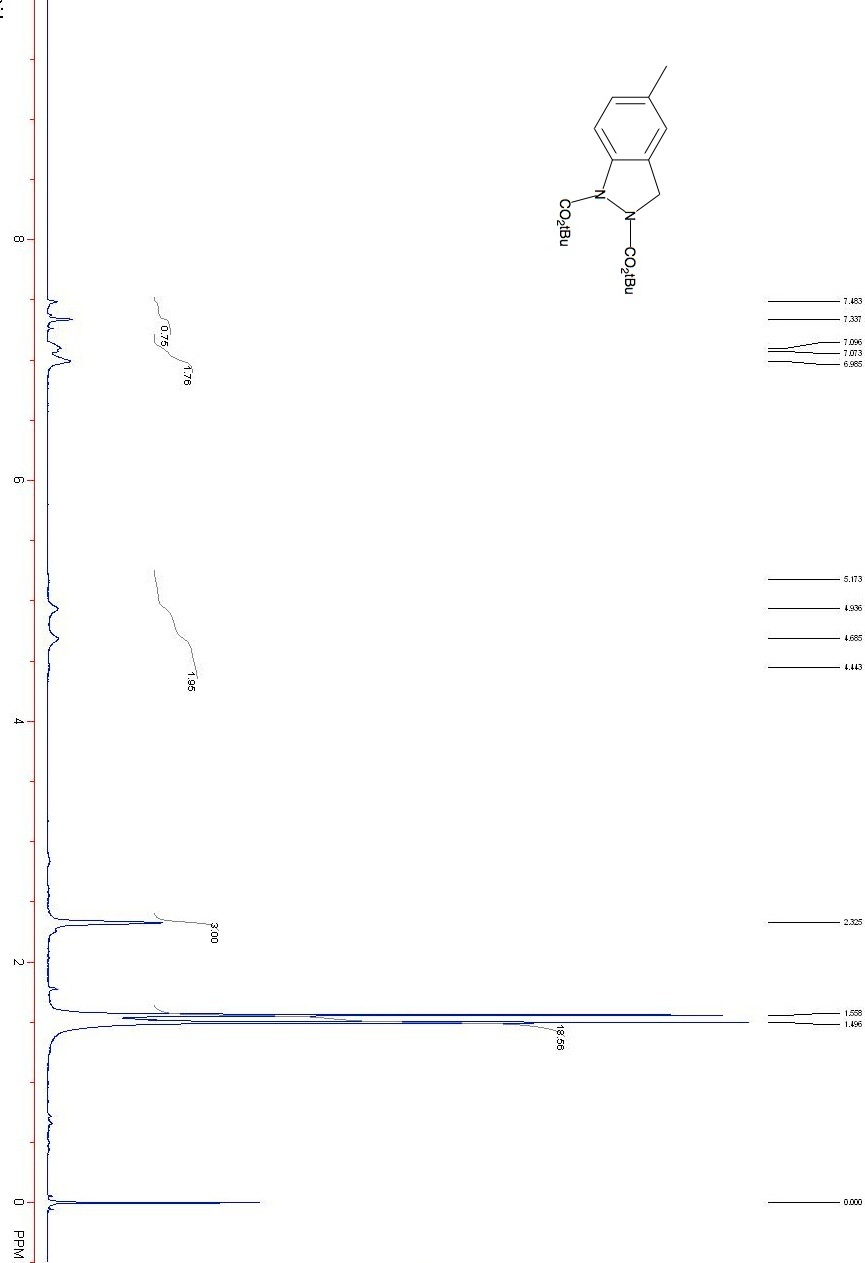
**

**8. ^13^C NMR spectrum of** **1,2-di-*tert*-butyl 5-methyl-1*H*-indazole-1,2-(3*H*)-dicarboxylate (10b)**

**
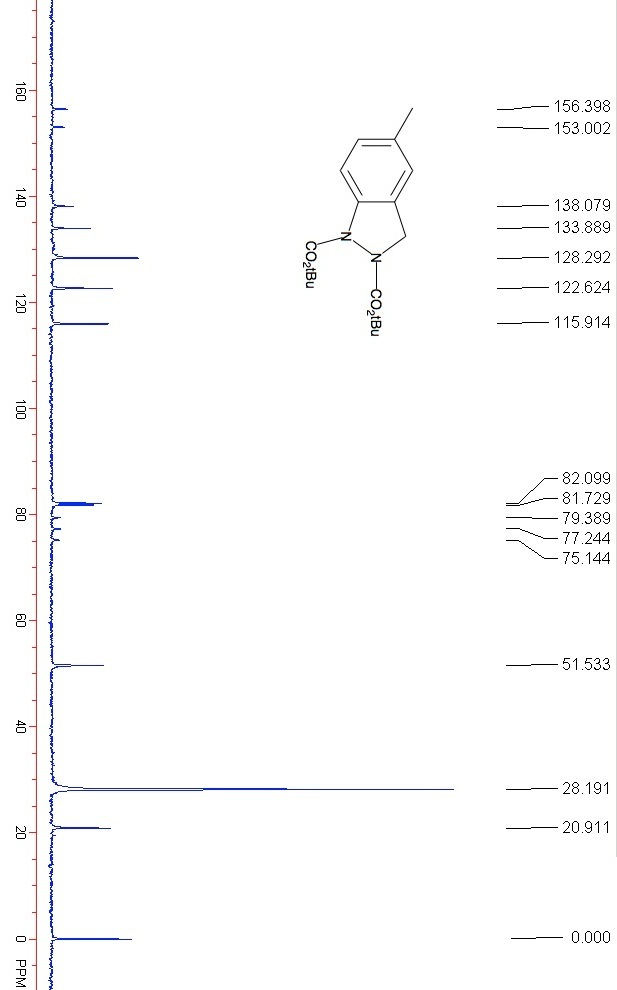
**

**9. ^1^H NMR spectrum of** **1,2-di-*tert*-butyl 5,6-dimethyl-1*H*-indazole-1,2-(3*H*)-dicarboxylate (10d)**

**
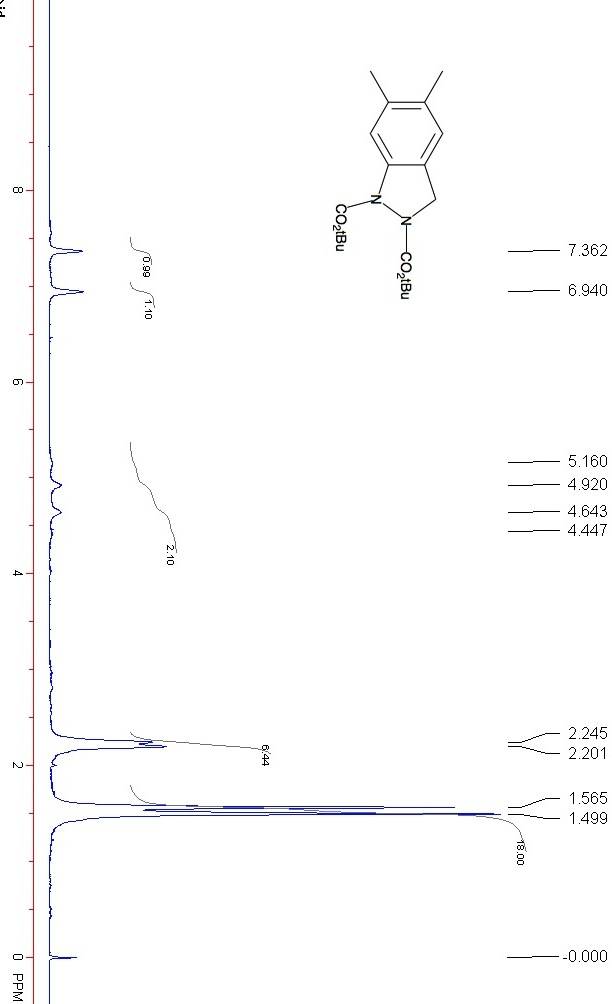
**

**10. ^13^C NMR spectrum of** **1,2-di-*tert*-butyl 5,6-dimethyl-1*H*-indazole-1,2-(3*H*)-dicarboxylate (10d)**

**
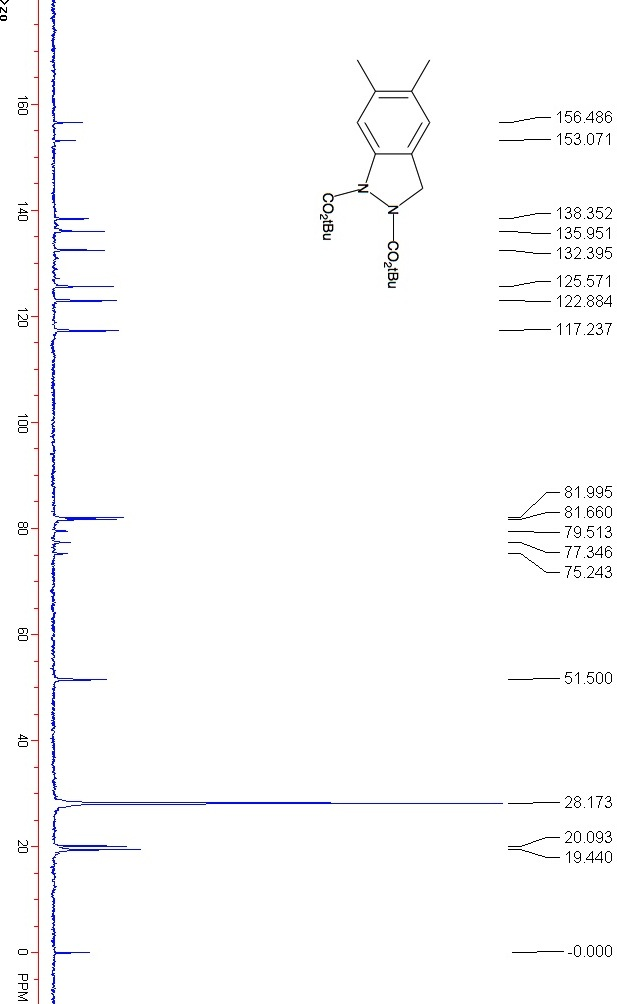
**

**11. ^1^H NMR spectrum of** **1,2-di-*tert*-butyl 5,6-dimethoxy-1*H*-indazole-1,2-(3*H*)-dicarboxylate (10e)**

**
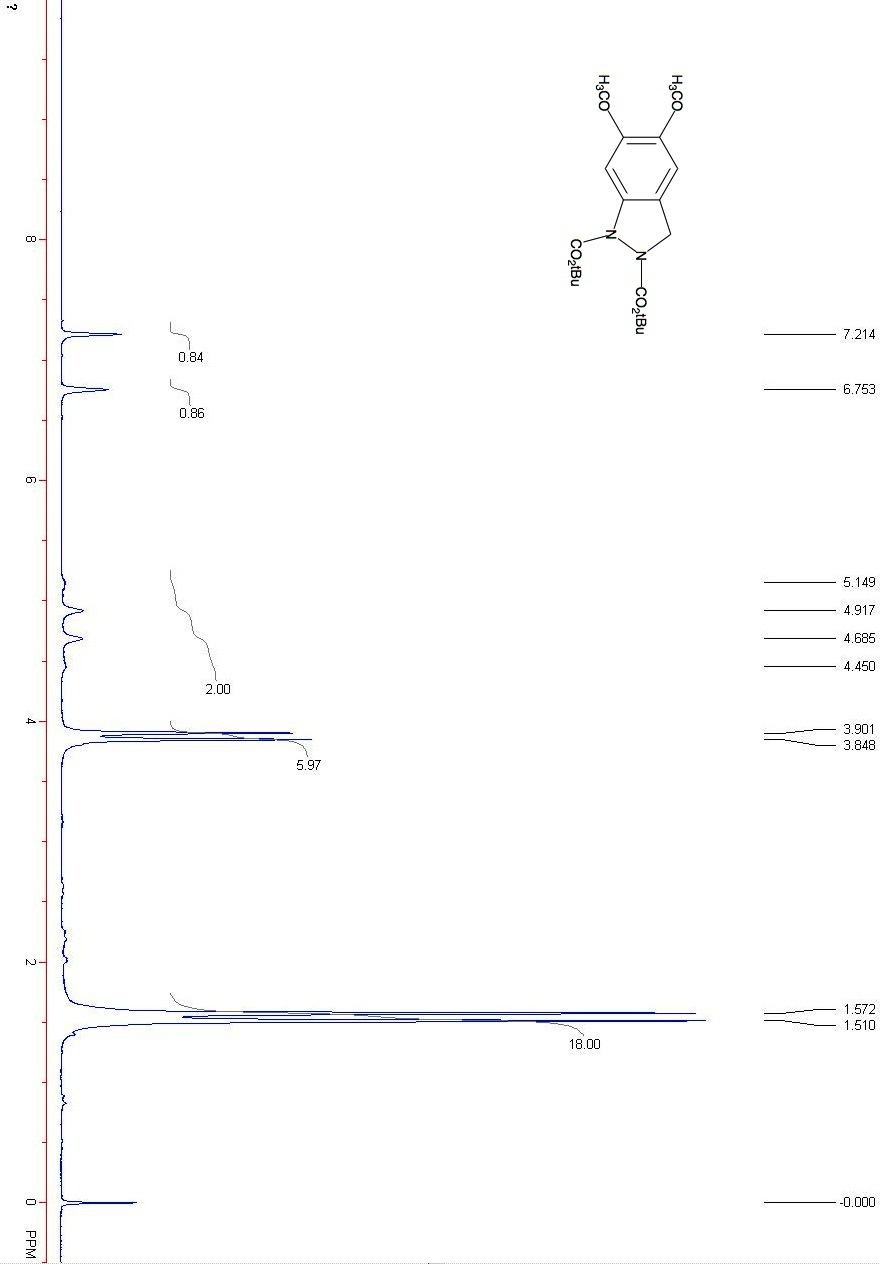
**

**12. ^13^C NMR spectrum of** **1,2-di-*tert*-butyl 5,6-dimethoxy-1*H*-indazole-1,2-(3*H*)-dicarboxylate (10e)**

**
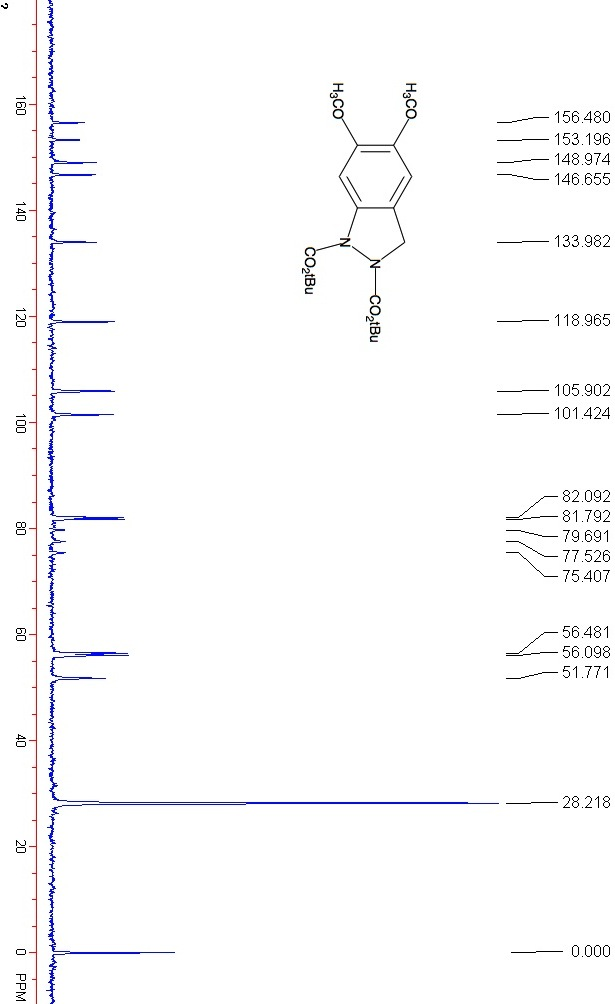
**
